# Effect of Neoadjuvant Therapy on Endoluminal Vacuum-Assisted Closure Therapy (EVAC) for Anastomotic Leakage After Oesophagectomy

**DOI:** 10.3390/cancers16213597

**Published:** 2024-10-25

**Authors:** Catharina Fahrenkrog, Sorin Miftode, Ahmed Al-Mawsheki, Fadl Alfarawan, Stella Wilters, Maximilian Bockhorn, Nader El-Sourani

**Affiliations:** 1 Universitätsmedizin Oldenburg, Carl von Ossietzky Universität Oldenburg, 26129 Oldenburg, Germany; 2 Universitätsklinik für Allgemein-und Viszeralchirurgie, Klinikum Oldenburg AöR, 26133 Oldenburg, Germany; 3 Klinik für Allgemein-, Viszeral-und Transplantationschirurgie, Universitätsklinikum Münster, 48149 Münster, Germany

**Keywords:** endoluminal vacuum-assisted closure, EVAC, anastomotic leakage, oesophagectomy, neoadjuvant therapy, chemotherapy, chemoradiation, oesophageal cancer

## Abstract

Background: Anastomotic leakage (AL) is a dreaded complication after oesophagectomy. Endoluminal vacuum-assisted closure therapy (EVAC) has been increasingly used as a first-line treatment for AL. We aimed to identify any potential adverse effects of a neoadjuvant therapy (chemotherapy (CT) or radiochemotherapy (RCT)) on EVAC. Methods: We performed a retrospective cohort study at our tertiary centre between 2013 and 2024. All patients who underwent EVAC for AL after oesophagectomy were included in this study. Parameters such as success rate, length of therapy, number of sponges needed, changes in treatment, and survival were analysed. Results: A total of 29 patients were included, 19 of whom received CT/RCT and 10 of whom received no neoadjuvant treatment (NT). There was no significant difference in patient survival (30-day survival rate CT/RCT *n* = 1 (5.3%) vs. NT *n* = 1 (10%), *p* = 0.632), success rate (CT/RCT *n* = 15 (78.9%) vs. NT *n* = 9 (90%), *p* = 0.454), and length of therapy (CT/RCT vs. NT 24.11 vs. 23.8, *p* = 0.681), the number of sponges required (CT/RCT vs. NT 6.26 vs. 6.6, *p* = 0.835), and the need for changing treatment (CT/RCT *n* = 5 (26.3%) vs. NT *n* = 1 (10%), *p* = 0.303). Conclusions: NT did not affect the success rate or length of therapy. Thus, we found no significant influence of CT/RCT on EVAC for AL after oesophagectomy.

## Simple Summary

Anastomotic leakage (AL) has a reported incidence of up to 53% and contributes to a high morbidity and mortality after oesophagectomy. Because of its high success rates, endoluminal vacuum-assisted closure therapy (EVAC) is nowadays the standard treatment for AL. However, its effectiveness depends upon different factors. As most patients receive trimodal therapy (neoadjuvant therapy, followed by surgery and adjuvant therapy) due to their preoperative cancer staging, the question arises as to whether neoadjuvant therapy impacts the success rate of EVAC. Therefore, our aim was to identify any adverse effects of a neoadjuvant therapy on EVAC compared to no prior treatment, in order to improve patient care and treatment algorithms. We found no significant influence of neoadjuvant therapy on EVAC. Furthermore, EVAC proved to be an effective and secure treatment option, leading to no changes in the management of AL for now.

## 1. Introduction

Anastomotic leakage (AL) is a common but much-dreaded complication after oesophagectomy [1]. In addition to AL, atrial fibrillation and urinary retention are other frequent postoperative complications [2], leading to a high number of readmissions, prolonged hospital stays, earlier relapses, and reduced health-related quality of life (HRQoL) [3,4]. Even though surgical techniques and patient care have improved, an oesophagectomy is associated with a high morbidity and mortality [2,5], not only posing a great risk factor to patients but also contributing to a high burden on the healthcare system [6]. Worldwide, oesophageal cancer is the eighth leading cause of cancer and contributes to a high number of cancer-associated deaths [7,8]. Because of its benefits in progression-free and long-term overall survival, a trimodal therapy of neoadjuvant treatment followed by surgical resection has been the standard of care [9]. All patients with oesophageal cancer stage cT3/T4 or N1-N3 receive a neoadjuvant treatment, either chemotherapy (CT) alone or combined radiochemotherapy (RCT), prior to an oesophageal resection. Although patient survival has improved, complications after oesophagectomy are still common, with reported rates between 17% and 74%, and AL being one of the most prevalent postoperative adverse events [1]. In 2015, the Esophagectomy Complications Consensus Group (ECCG) evaluated 2704 patients who underwent oesophagectomy and found that 11.4% developed AL [1]. Other studies report incidences as high as 53% [10,11]. Depending on the extent of the AL, conservative (antibiotics, nil by mouth, and gastric drainage), endoscopic (stents or vacuum-assisted closure), or surgical interventions can be considered as treatment [12]. EVAC has been increasingly used in the treatment of AL. The continuous negative pressure applied to the lesion guarantees drainage of the cavity, while also controlling oedema, promoting granulation, and reducing contamination of the defect [4,13]. Its treatment success has been reported between 66.7% and 100% and explains the widespread use at present [13]. With neoadjuvant therapy not only targeting malignant cells but also affecting healthy adjacent tissue [14], the question arises whether the success of EVAC for AL after oesophagectomy is affected by prior CT or RCT. Despite this common knowledge, Seika et al. are the only ones to investigate a possible effect on EVAC for AL to this day. They described a significant influence of a neoadjuvant therapy on the outcome of EVAC for AL after oesophagectomy [15]. They showed that the duration of EVAC in patients with neoadjuvant RCT was significantly longer and was associated with more interventions than in patients with neoadjuvant CT [15]. There are a few studies, however, concerning this issue in rectal anastomotic leakage with heterogeneous results. While one study found a correlation between neoadjuvant therapy and EVAC for AL, the other one found no influence on EVAC [16,17]. As a possible correlation could have consequences on future treatment decisions, and with the aim of improving patient care and treatment algorithms, we aimed to further identify any adverse effects of neoadjuvant therapy in general (either CT or combined RCT) on EVAC treatment of AL compared to no prior treatment. 

## 2. Materials and Method

### 2.1. Study Design and Participants

**Figure 1:**
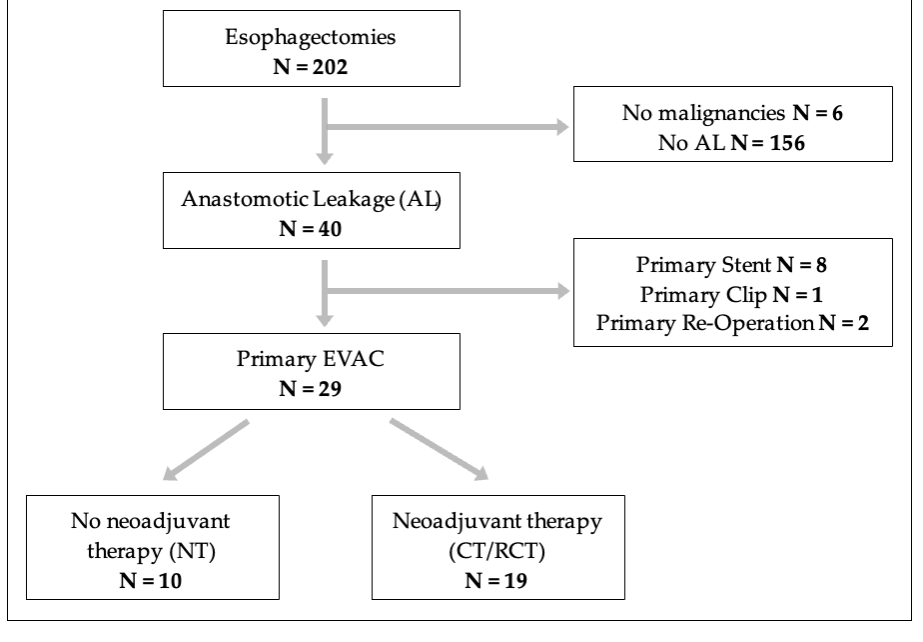
Flow-chart showing the study enrolment.

A retrospective cohort study was performed at our tertiary centre and included all patients undergoing EVAC therapy for AL after oesophagectomy for underlying cancer between 2013 and 2024. Patients with no malignancies or those who did not receive EVAC therapy were excluded. Patients were compared based on whether they had received a neoadjuvant therapy (chemotherapy or combined radiochemotherapy (CT/RCT)) or no prior treatment before oesophagectomy. Figure 1 depicts the study population.

### 2.2. Primary and Secondary Outcomes

The success rate of EVAC therapy, defined as complete closure of the defect, was set as the primary outcome. The duration of EVAC, number of sponges needed, and change in treatment modality, as well as the 30-day and 90-day mortality, were analysed as secondary outcomes. 

### 2.3. Statistical Analysis

The statistical analysis was performed using the Statistical Package for the Social Sciences (SPSS), version 29.0.2.0. Pre- and perioperative patient characteristics were analysed using standard descriptive measurements. The results were expressed as means with standard deviation (SD) for continuous variables, or as counts and percentages for categorial variables. A two-sample *t*-test was used to compare the differences between means and Pearson’s Chi-squared test for differences between counts. A *p* ≤ 0.05 was considered statistically significant. To measure the effect size, Cohen’s d and Phi were used for continuous variables and categorial variables, respectively. The post hoc power of the analysis was calculated using G*Power 3.1 [18]. 

### 2.4. Preoperative Assessment

All patients received a routine evaluation as part of the preoperative preparation. This included medical history, physical examination, and laboratory and imaging studies, as well as anaesthetic assessment. Patients were admitted to the hospital for an elective oesophagectomy after diagnosis and staging of oesophageal cancer and, if required, neoadjuvant therapy was completed in an out-clinic setting. Diagnosis and staging were based on endosonography, oesophagogastroduodenoscopy, and cross-sectional imaging via computed tomography of the chest and abdomen. All patients were presented and discussed in a multidisciplinary tumour board. 

### 2.5. Neoadjuvant Treatment

If indicated, patients either received chemotherapy (CT) following the FLOT regime [19] or combined radiochemotherapy (RCT) using the CROSS protocol [20] prior to oesophagectomy. The necessity of neoadjuvant therapy was determined by discussing the individual case in a multidisciplinary tumour board, according to treatment recommendations and current standards at that time. 

### 2.6. Surgical Procedure

Patients underwent either an open, hybrid, totally laparoscopic, or robotic-assisted surgical approach. The procedure was performed according to national and international standards. All patients were operated on by the same two surgeons using a two-stage approach, consisting of an abdominal and thoracic phase. Patients received an Ivor Lewis oesophagectomy with a gastric conduit and a standardised two-field lymphadenectomy. One patient required a colon interposition due to a previous Billroth-II resection. Once gastric integrity was reestablished, a nasogastric tube was placed into the gastric conduit, and a pleural drain was inserted before wound closure. 

### 2.7. Postoperative Management

After surgery, all patients were admitted to our surgical intensive care unit (ICU) and, once stabilised, transferred to the intermediate care unit (IMC) before being admitted to the surgical ward. Enteral feeding was administered through the nasogastric tube. On day five, the nasogastric tube was removed, and oral intake was allowed. Routine examination of the anastomosis was not performed. In the case of symptoms such as fever, pain, or new onset of atrial arrhythmia, a combination of upper endoscopy and computed tomography was performed. If an AL was present, EVAC was initiated. AL, conduit necrosis, pneumonia, acute respiratory distress syndrome (ARDS), wound infections, and organ failure were reported as postoperative complications. In addition, mortality was recorded and divided into 30- and 90-day mortality. All postoperative complications were graded using the Clavien–Dindo classification of surgical complications [21]. 

### 2.8. Endoluminal-Vacuum-Assisted Therapy (EVAC) 

EVAC was performed after diagnosis of AL via gastroscopy or computer tomography. Postoperative imaging was conducted if patients presented with either newly elevated or persistently high inflammation markers, detectable nutrition or stomach contents in pleural drainage, or new onset of atrial arrhythmia. The decision for EVAC was made interdisciplinarily by an internal medicine endoscopist and a visceral surgeon. The sponge for EVAC was placed into the abscess cavity or left in an intraluminal position to cover the leak in the case of small defects. The vacuum pressure was individually regulated between 75 and 150 mmHg of continuous pressure and adjusted throughout the course. With sponge changes every three days, the defect was reassessed. Once the defect was closed and sufficient granulation tissue was present, EVAC was discontinued. If patients presented with symptoms of AL after EVAC discontinuation, endoscopy was performed again. In case of an AL, EVAC was restarted. In the event of no improvement under EVAC or further deterioration of the patient’s condition, other treatment modalities were discussed interdisciplinarily. In cases of a persisting leak, necrosis, or ischemia of the conduit, surgical revision was required. 

### 2.9. Ethical Approval

Under the terms of §15 BO ÄKN, a request for ethical approval was submitted to the responsible ethics committee of the Carl von Ossietzky University of Oldenburg. This study was conducted in accordance with the Declaration of Helsinki, and the protocol was approved by the ethics committee (AZ-2024-087).

## 3. Results

### 3.1. Patients’ Characteristics 

A total of 196 patients received oesophagectomy for oesophageal cancer between 2013 and 2024, as shown in Figure 1. Out of the 196 patients, 112 had a neoadjuvant therapy, and of those patients, 19 (16.96%) developed an AL. Overall, 29 patients presented with AL and were included in our final analysis, with 19 having received a neoadjuvant therapy (CT/RCT) and 10 having received no neoadjuvant therapy (NT). The baseline characteristics are shown in Table 1.

Patients in the NT group were older than those in the CT/RCT group (NT 71.3 years vs. CT/RCT 61.05 years, *p* = 0.046, with an effect size Cohen’s d = 0.772 and post hoc power of 0.61). In addition, preoperatively more patients in the NT group presented with arterial hypertension and hyperlipidaemia (NT *n* = 9 (90%) vs. CT/RCT *n* = 10 (52.6%), *p* = 0.044, phi = −0.374 and NT *n* = 2 (10%) vs. CT/RCT *n* = 0 (0%), *p* = 0.043, phi = −0.375, respectively). There was a trend towards more coronary heart disease in the NT group (NT *n* = 3 (30%) vs. CT/RCT *n* = 1 (5.3%), *p* = 0.066, phi = −0.341) and more patients with a pulmonary disease in the CT/RCT group (CT/RCT *n* = 3 (15.8%) vs. NT *n* = 0 (0%), *p* = 0.184, phi = 0.246). Comorbidities are presented in Table 2. 

### 3.2. Histopathological Characteristics

The pre- and postoperative histopathological gradings are shown in Table 3. No significant differences were distinguishable. 

### 3.3. Postoperative Complications

Table 4 displays the postoperative morbidity and mortality. There was no difference in 30- and 90-day mortality between the NT and CT/RCT groups (NT *n* = 1 (10%) vs. CT/RCT *n* = 1 (5.3%), *p* = 0.632, with an effect size phi = −0.089 and NT *n* = 1 (10%) vs. CT/RCT *n* = 4 (21.1%), *p* = 0.454, phi = 0.139, respectively). Similarly, the incidence of postoperative complications, as well as their classification (Clavien–Dindo), did not differ. 

### 3.4. Anastomotic Leakage and EVAC

Characteristics of anastomotic leakage and its treatment with EVAC are presented in Table 5. There was no significant difference in the success rate of EVAC (NT *n* = 9 (90%) vs. CT/RCT *n* = 15 (78.9%), *p* = 0.454, with an effect size phi = −0.139), the time from oesophageal resection until AL diagnosis (NT 12.6 days vs. CT/RCT 10.9 days, *p* = 0.589, with an effect size Cohen’s d = 0.216), the duration of EVAC therapy (NT 23.8 days vs. CT/RCT 24.1 days, *p* = 0.964, Cohen’s d = 0.018), and the number of sponges needed until defect closure (NT 6.6 vs. CT/RCT 6.3, *p* = 0.835, Cohen’s d = 0.085) between the two groups. Furthermore, there was no statistically significant influence on the need for transition to stent therapy (NT *n* = 1 (10%) vs. CT/RCT *n* = 5 (26.3%), *p* = 0.303, phi = 0.191) and the operative revision rate (NT *n* = 0 (0%) vs. CT/RCT *n* = 1 (5.3%), *p* = 0.46, phi = 0.137). 

## 4. Discussion

In this study, we analysed if a neoadjuvant therapy has any adverse effects on EVAC treatment for AL after an oesophagectomy compared to no prior treatment. Neoadjuvant therapy is either conducted as chemotherapy (CT) alone or combined radiochemotherapy (RCT), with the decision depending on the tumour aetiology, condition, age, and comorbidities of the patient [9]. While the toxicity of chemotherapy is achieved by interrupting the synthesis of RNA and DNA [14], irradiation induces breaks in already present DNA, causing the termination of cell division and proliferation [22]. Through apoptosis, these mechanisms reduce the tumour size and existing metastases, leading to significantly improved locoregional and distant disease control, with enhanced resectability and survival [20,23]. Although the aim is to only target malignant cells with neoadjuvant therapy, affecting healthy tissue is inevitable. As the ordered sequence of cellular mechanisms is disrupted, repetitive inflammatory responses and cellular regeneration are triggered, with the possibility of uncontrolled matrix accumulation and fibrosis [24]. This might further contribute to the already difficult healing conditions of a subsequent anastomosis after an oesophagectomy and could explain longer treatment durations, more interventions, and more sponge exchanges. However, opposing results can be found in the literature. Several studies suggest that the addition of radiation is associated with a higher morbidity and mortality and have reported an increased risk of AL if the anastomosis is placed in the radiation field [25], while others have found no influence of neoadjuvant therapy on AL incidence [26,27], and others again reported a correlation between the radiation dose or location and the incidence of AL [28,29,30]. Even though the incidence of AL varies across different studies, it is clear that AL contributes to a high morbidity and mortality. The role of neoadjuvant treatment and other risk factors in its onset has been broadly investigated, with similarly diverse findings [31,32]. How the treatment of AL after oesophagectomy is affected by neoadjuvant therapy has been less studied. So far, Seika et al. are, to the best of our knowledge, the only ones examining a possible correlation. They compared the efficacy of endoluminal vacuum-assisted therapy for AL in a total of 26 patients, with 13 having received neoadjuvant CT and 13 neoadjuvant RCT [15]. They found a significantly longer treatment duration of EVAC in the RCT group (CT 14.69 days vs. RCT 20.85 days, *p* = 0.002), with a significantly higher number of endoscopic interventions and consequently more sponges needed (CT *n* = 4.38 vs. RCT *n* = 6.85, *p* = 0.001), and suggested irradiation as a possible explanation [15]. Since the benefits of any neoadjuvant therapy on the survival of patients with oesophageal cancer have become apparent, it has been firmly implemented in standardised treatment guidelines. This is why we decided to compare the general use of neoadjuvant therapy with no neoadjuvant therapy at all on EVAC for AL, unlike Seika et al. Thus, we compared a group of patients who received either chemotherapy or combined radiochemotherapy (CT/RCT) with another group of patients who received no prior treatment (NT) at all before oesophagectomy. In contrast to Seika et al., our study showed no significant influence of CT/RCT on the outcomes of EVAC for anastomotic leakages. Even though there is practically no literature concerning the effect of neoadjuvant therapy on EVAC for AL after oesophagectomy, a few studies have investigated this issue in rectal anastomotic leakage. Generally, patients were also treated for cancer with either neoadjuvant therapy or no prior treatment before rectal resection followed by reconstruction of the rectum by forming an anastomosis, making it comparable to our study. Bernstoff et al. showed that not only was the duration of EVAC significantly longer in patients with neoadjuvant therapy, but patients also had significantly more sponge exchanges and endoscopies until defect closure [16]. On the contrary, Strobel et al. found no influence of neoadjuvant therapy on EVAC [17]. 

Our success rate of primary EVAC in the NT group of 80% and in the CT/RCT group of 57.9% (*p* = 0.234) is similar to the reported treatment success, ranging from 66.7% to 100% in the literature [13], and further supports the already known benefits of EVAC in AL treatment. The success rate is the most crucial outcome for EVAC. Parameters such as the length of therapy, number of sponges, and changes in treatment modality are likewise important aspects in the short-term follow-up. EVAC has proven to be a sufficient treatment option in the management of AL, as it continuously drains fluids and edema, reduces bacterial contamination, increases vascularity, and promotes the formation of granulation tissue, thereby facilitating wound closure [13,31,33]. Even though there is growing evidence for the success of EVAC in the literature, there are still no specific treatment guidelines [34]. As most clinics do not follow a standardised protocol in EVAC practice, the comparison of different studies is complicated and can only be made with caution. This also applies to our study. Without a standardised guideline to follow, and still in unison with the current literature, the pressure of vacuum therapy was chosen individually during endoscopy, making it dependent on the decision of the consulting physician. Nevertheless, our findings can still serve as guidance for other physicians performing EVAC, as EVAC has been well established in the treatment of AL over the last decade and is internationally considered the gold standard [12,13,35].

Verstegen et al. performed a systematic literature search for the management of AL and compared 19 different studies with 273 patients in total. Because of the heterogeneity of the included studies, no uniform treatment recommendation could be gathered, and an individual approach for each patient was advised [36]. Furthermore, they recommended using a grading system of AL based on the characteristics of the AL itself, rather than the treatment of AL, as is currently implemented with the ECCG system. The ECCG system classifies AL based on the performed treatment and cannot therefore be used for treatment guidelines at first. The disparity between scoring systems and their implementation in practice might further contribute to the heterogeneity of the literature, hindering the development of uniform guidelines and leaving the management of AL complicated. AL remains an interdisciplinary challenge for surgeons and other specialties involved in treatment and patient care, even without considering neoadjuvant therapy and its impact on EVAC. As AL is significantly correlated with longer hospital stays, more reoperations, and higher morbidity [35], it is important to improve the diagnosis and management of AL to alleviate high costs and the burden on the healthcare system. With EVAC being the treatment of choice for AL today, and with the scarce and heterogeneous literature concerning the effects of neoadjuvant therapy on EVAC in mind, further studies must be performed to especially investigate possible correlations in this regard. 

### Limitations

Our study is limited by its small sample size and retrospective study design. To further support the results and to allow the generation of standardised treatment guidelines, a multicentre analysis is inevitable. Even though AL is a common complication after oesophagectomy, gathering enough patients to provide robust data is a long-lasting and time-consuming endeavour, thus leading to a small sample size. During this timeline, treatment adjustments or modifications often take place, leaving the obtained results questionable. Our study reached back 10 years and could still include only 29 patients, which demonstrates the previously mentioned challenge. We performed a post hoc power analysis and an analysis of the effect size to balance for the small sample size and to alleviate the interpretation of the results. Furthermore, the differences in age and comorbidities between the two treatment groups must be pointed out. Patients who received no prior treatment to oesophagectomy were significantly older than patients with neoadjuvant therapy. They were also more often diagnosed with arterial hypertension and hyperlipidaemia. Even though there was no significant difference in other comorbidities, their existence and the older age of the patients might restrict the possibility of neoadjuvant therapy in some cases. Without the potential downsizing of the tumour and the benefits of neoadjuvant therapy, patients are often left more prone to a worsened course of their health, making them possibly more susceptible to postoperative complications. As AL is a significant complication in the early postoperative period, our interest focused on short-term outcomes. Thus, any possible long-term complications of AL, such as HRQoL or stenosis of the anastomosis, were not included in this study.

Despite the mentioned limitations, this study still provides relevant results. It confirms the efficacy of endoluminal vacuum-assisted therapy in the treatment of anastomotic leakages and further reinforces the need for larger sample sizes and standardized treatment guidelines.

## 5. Conclusions

In this study, there was no significant influence of neoadjuvant therapy on EVAC for anastomotic leakages after oesophagectomy, leading to no changes in the management of AL for now. As this cohort was comparatively small, further research must be performed to validate these results. Nonetheless, EVAC proved to be an effective and secure treatment option, even if patients may require longer hospital stays. Since AL still poses an interdisciplinary challenge, it is important to continuously reassess even well-established procedures with the aim of constant improvement and the maintenance of high standards to ensure patient safety.

## Figures and Tables

**Table 1:** Baseline characteristics.

		Total (*n* = 29)	NT (*n* = 10)	CT/RCT (*n* = 19)	*p*-Value (95% CI *)	Effect Size	
						Cohen’s d	Phi
Sex, n (%)					0.5 ^a^ (−0.189–0.379)	-	−0.131
	Male	25 (86.2)	8 (80)	17 (89.5)			
	Female	4 (13.8)	2 (20)	2 (10.5)			
Age at resection, mean (SD)		64.6 (±13.3)	71.3 (±9.8)	61.1 (±13.7)	0.046 ^b^ (0.198–20.296)	0.772	-
BMI ** in kg/m^2^, mean (SD)		25.3 (±4.8)	26 (±4.4)	25 (±5.1)	0.603 ^b^ (−2.897–4.897)	0.208	-
Operation type, n (%)					0.054 ^a^ (−0.006–0.764)	-	−0.362
	Open	13 (44.8)	2 (20)	11 (57.9)			
	Laparoscopic	16 (55.2)	8 (80)	8 (42.1)			
Operation time in minutes, mean (SD)		265.5 (±82.8)	249.1 (±74.7)	274.1 (±87.4)	0.449 ^b^ (−91.849–41.838)	0.302	-
ASA *** Score, n (%)					0.583 ^a^ (−0.340–0.593)	-	0.243
	1	0 (0)	0 (0)	0 (0)			
	2	15 (51.7)	4 (40)	11 (57.9)			
	3	13 (44.8)	6 (60)	7 (36.8)			
	4	1 (3.4)	0 (0)	1 (5.3)			
	5	0 (0)	0 (0)	0 (0)			
	6	0 (0)	0 (0)	0 (0)			
Length of hospital stay in days, mean (SD)		64.5 (±37.9)	59.5 (±36.9)	67.1 (±39.1)	0.618 ^b^ (−38.303–23.198)	0.199	-
Length of combined stay at ICU **** and IMC ^†^ in days, mean (SD)		40.5 (±40.1)	33.1 (±35.9)	44.4 (±42.5)	0.482 ^b^ (−43.675–21.138)	0.281	-
Blood transfusions during hospital stay, mean (SD)		1.7 (±4.9)	1.9 (±3.7)	1.5 (±5.5)	0.850 ^b^ (−3.630–4.378)	0.075	-

* CI = confidence interval, ^a^ = Pearson’s Chi-squared test, the Chi-squared statistics are significant at a level of 0.05, ^b^ = two-sample *t*-test for difference of means, the *t*-test statistics are significant at a level of 0.05, ** = body mass index, *** = American Society of Anesthesiologists, **** = Intensive Care Unit, ^†^ = Intermediate Care Unit.

**Table 2:** Preoperative comorbidities.

		Total (*n* = 29)	NT (*n* = 10)	CT/RCT (*n* = 19)	*p*-Value	Effect Size
						Phi
Comorbidities, *n* (%)						
	Cardiovascular	22 (75.9)	9 (90)	13 (68.4)	0.197 ^a^	−0.240
	Arterial hypertension	19 (65.5)	9 (90)	10 (52.6)	0.044 ^a^	−0.374
	Coronary heart disease	4 (13.8)	3 (30)	1 (5.3)	0.066 ^a^	−0.341
	Heart failure (decreased EF)	1 (3.4)	0 (0)	1 (5.3)	0.460 ^a^	0.137
	Cardiac arrhythmia	4 (13.8)	1 (10)	3 (15.8)	0.667 ^a^	0.080
	Pulmonary	3 (10.3)	0 (0)	3 (15.8)	0.184 ^a^	0.246
	COPD	1 (3.4)	0 (0)	1 (5.3)	0.460 ^a^	0.137
	Bronchial asthma	1 (3.4)	0 (0)	1 (5.3)	0.460 ^a^	0.137
	Renal	1 (3.4)	0 (0)	1 (5.3)	0.460 ^a^	0.137
	Diabetes	5 (17.2)	1 (10)	4 (21.1)	0.454 ^a^	0.139
	Obesity (BMI ≥ 30)	5 (17.2)	1 (10)	4 (21.1)	0.454 ^a^	0.139
	Hyperlipidaemia	2 (6.9)	2 (20)	0 (0)	0.043 ^a^	−0.375
	Alcohol abuse	2 (6.9)	0 (0)	2 (10.5)	0.288 ^a^	0.197
	Nicotine abuse	7 (24.1)	2 (20)	5 (26.3)	0.706 ^a^	0.070

^a^ = Pearson’s Chi-squared test, the Chi-squared statistics are significant at a level of 0.05.

**Table 3:** Pre- and postoperative grading.

			Total (*n* = 29)	NT (*n* = 10)	CT/RCT (*n* = 19)	*p*-Value	Effect Size
							Phi
Aetiology, *n* (%)						0.161 ^a^	−0.260
	Adenocarcinoma		28 (96.6)	9 (90)	19 (100)		
	Squamous cell carcinoma		1 (3.4)	1 (10)	0 (0)		
Preoperative staging, n (%)							
	Tumour size (cT)	T1	2 (6.9)	1 (10)	1 (5.3)	0.204 ^a^	0.331
		T2	9 (31)	5 (50)	4 (21.1)		
		T3	18 (62.1)	4 (40)	14 (73.7)		
		T4	0 (0)	0 (0)	0 (0)		
	Nodular involvement (cN)	N0	10 (34.5)	5 (50)	5 (26.3)	0.564 ^a^	0.320
		N1	5 (17.2)	2 (20)	3 (15.8)		
		N2	2 (6.9)	0 (0)	2 (10.5)		
		N3	1 (3.4)	0 (0)	1 (5.3)		
		+	11 (37.9)	3 (30)	8 (42.1)		
	Metastatic spread (cM)	M0	26 (89.7)	10 (100)	16 (84.2)	0.415 ^a^	0.246
		M1	1 (3.4)	0 (0)	1 (5.3)		
		Mx	2 (6.9)	0 (0)	2 (10.5)		
Postoperative staging, *n* (%)							
	Tumour size (cT)	T0	6 (20.7)	1 (10)	5 (26.3)	0.307 ^a^	0.407
		T1	7 (24.1)	4 (40)	3 (15.8)		
		T2	3 (10.3)	2 (20)	1 (5.3)		
		T3	12 (41.4)	3 (30)	9 (47.4)		
		T4	1 (3.4)	0 (0)	1 (5.3)		
	Nodular involvement (cN)	N0	16 (55.2)	7 (70)	9 (47.4)	0.346 ^a^	0.338
		N1	5 (17.2)	2 (20)	3 (15.8)		
		N2	3 (10.3)	1 (10)	2 (10.5)		
		N3	5 (17.2)	0 (0)	5 (26.3)		
	Metastatic spread (cM)	M0	27 (93.1)	10 (100)	17 (89.5)	0.288 ^a^	0.197
		M1	2 (6.9)	0 (0)	2 (10.5)		
	Differentiation (G)	G1	1 (3.4)	1 (10)	0 (0)	0.549 ^a^	0.266
		G2	9 (31)	4 (40)	5 (26.3)		
		G3	7 (24.1)	3 (30)	4 (21.1)		
		G4	0 (0)	0 (0)	0 (0)		
		Missing	12 (41.4)	2 (20)	10 (52.6)		
	Lymphatic invasion (L)	L0	17 (58.6)	8 (80)	9 (47.4)	0.216 ^a^	0.243
		L1	9 (31)	2 (20)	7 (36.8)		
		Missing	3 (10.3)	0 (0)	3 (15.8)		
	Vascular invasion (V)	V0	26 (89.7)	10 (100)	16 (84.2)	-	-
		V1	0 (0)	0 (0)	0 (0)		
		Missing	3 (10.3)	0 (0)	3 (15.8)		
	Perineural invasion (Pn)	Pn0	29 (100)	10 (100)	19 (100)	-	-
		Pn1	0 (0)	0 (0)	0 (0)		
	Resection margins (R)	R0	28 (96.6)	10 (100)	18 (94.7)	0.460 ^a^	0.137
		R1	1 (3.4)	0 (0)	1 (5.3)		
		R2	0 (0)	0 (0)	0 (0)		

^a^ = Pearson’s Chi-squared test, the Chi-squared statistics are significant at a level of 0.05.

**Table 4:** Postoperative morbidity and mortality after oesophagectomy.

			Total (*n* = 29)	NT (*n* = 10)	CT/RCT (*n* = 19)	*p*-Value (95% CI *)	Effect Size	
							Cohen’s d	Phi
30-day mortality, *n* (%)			2 (6.9)	1 (10)	1 (5.3)	0.632 ^a^	-	−0.089
90-day mortality, *n* (%)			5 (17.2)	1 (10)	4 (21.1)	0.454 ^a^	-	0.139
CRP in mg/dL 3rd postoperative day, mean (SD)			21.2 (±8.9)	22.6 (±9.5)	20.5 (±8.7)	0.568 ^b^ (−5.362–9.567)	0.237	-
CRP in mg/dL 5th postoperative day, mean (SD)			22.3 (±9.9)	22.9 (±6.4)	22.0 (±11.6)	0.839 ^b^ (−7.314–8.939)	0.082	-
CRP in mg/dL 8th postoperative day, mean (SD)			20.2 (±10.2)	23.0 (±10.6)	18.8 (±9.9)	0.292 ^b^ (−3.865–12.368)	0.419	-
Total, *n* (%)			19 (65.5)	7 (70)	12 (63.2)	0.713 ^a^	-	−0.068
	Conduit necrosis		0 (0)	0 (0)	0 (0)	-	-	-
	Pneumonia		16 (55.2)	5 (50)	11 (57.9)	0.684 ^a^	-	0.075
	ARDS		13 (44.8)	6 (60)	7 (36.8)	0.233 ^a^	-	−0.221
Clavien–Dindo classification, *n* (%)						0.813 ^a^	-	0.358
	I		0 (0)	0 (0)	0 (0)			
	II		0 (0)	0 (0)	0 (0)			
		IIa	1 (3.4)	0 (0)	1 (5.3)			
	III		1 (3.4)	0 (0)	1 (5.3)			
		IIIa	14 (48.3)	5 (50)	9 (47.4)			
		IIIb	6 (20.7)	2 (20)	4 (21.1)			
	IV		1 (3.4)	1 (10)	0 (0)			
		IVa	1 (3.4)	0 (0)	1 (5.3)			
		IVb	3 (10.3)	1 (10)	2 (10.5)			
	V		2 (6.9)	1 (10)	1 (5.3)			

* CI = confidence interval, ^a^ = Pearson’s Chi-squared test, the Chi-squared statistics are significant at a level of 0.05, ^b^ = two-sample *t*-test for difference of means, the *t*-test statistics are significant at a level of 0.05.

**Table 5:** Anastomotic leakage (AL) and EVAC therapy.

		Total (*n* = 29)	NT (*n* = 10)	CT/RCT (*n* = 19)	*p*-Value (95% CI *)	Effect Size	
						Cohen’s d	Phi
Diagnosis of AL **, *n* (%)					0.215 ^a^	-	0.326
	Gastroscopy	14 (48.3)	3 (30)	11 (57.9)			
	CT	1 (3.4)	0 (0)	1 (5.3)			
	CT + gastroscopy	14 (48.3)	7 (70)	7 (36.8)			
Days to diagnosis, mean (SD)		11.5 (±7.6)	12.6 (±9.8)	10.9 (±6.5)	0.589 ^b^ (−4.544–7.849)	0.216	-
Initial defect size of AL in mm, mean (SD)		17.2 (±22.4)	16.3 (±10.7)	17.5 (±26.1)	0.896 ^b^ (−21.11–18.557)	0.057	-
Localisation of AL from the row of teeth in cm, mean (SD)		27.8 (±13.5)	29.0 (±3.2)	27.1 (±3.8)	0.198 ^b^ (−1.038–4.775)	0.509	-
ECCG *** leak classification, *n* (%)					0.118 ^a^	-	0.290
	I	0 (0)	0 (0)	0 (0)			
	II	25 (86.2)	10 (100)	15 (78.9)			
	IIIa	4 (13.8)	0 (0)	4 (21.1)			
	IIIb	0 (0)	0 (0)	0 (0)			
Successful defect closure, *n* (%)		24 (82.8)	9 (90)	15 (78.9)	0.454 ^a^	-	−0.139
Successful primary EVAC ^†^, *n* (%)		19 (65.5)	8 (80)	11 (57.9)	0.234 ^a^	-	−0.221
Length of EVAC in days, mean (SD)		24 (±17.0)	23.8 (±12.2)	24.11 (±19.4)	0.964 ^b^ (−14.211–13.601)	0.018	-
Number of sponges needed, mean (SD)		6.4 (±4.0)	6.6 (±3.34)	6.3 (±4.4)	0.835 ^b^ (−2.943–3.617)	0.085	-
EVAC failure		10 (34.5)	5 (50)	5 (26.3)			
	Change to stent, *n* (%)	6 (20.7)	1 (10)	5 (26.3)	0.303 ^a^	-	0.191
	Number of stents, mean (SD)	0.3 (±0.8)	0.3 (±0.9)	0.4 (±0.8)	0.834 ^b^ (−0.732–0.595)	0.086	-
	Usage of clips, *n* (%)	1 (3.4)	0 (0)	1 (5.3)	0.460 ^a^	-	0.137
	Number of clips needed, mean (SD)	0.03 (±0.2)	0 (±0)	0.05 (±0.2)	0.478 ^b^ (−0.203–0.098)	0.269	-
	Surgical revision, *n* (%)	1 (3.4)	0 (0)	1 (5.3)	0.460 ^a^	-	0.137

* CI = confidence interval, ^a^ = Pearson’s Chi-squared test, the Chi-squared statistics are significant at a level of 0.05, ^b^ = Two-sample *t*-test for difference of means, the *t*-test statistics are significant at a level of 0.05, ** = Anastomotic leakage, *** = Esophageal Complications Consensus Group, ^†^ = Endoluminal-vacuum-assisted therapy.

## Data Availability

The original contributions presented in this study are included in the article material. Further inquiries can be directed to the corresponding author.
